# Postural orientation, what to expect in youth athletes? A cohort study on data from the Malmö Youth Sport Study

**DOI:** 10.1186/s13102-021-00307-y

**Published:** 2021-07-24

**Authors:** Sofia Ryman Augustsson, Jenny Nae, Magnus Karlsson, Tomas Peterson, Per Wollmer, Eva Ageberg

**Affiliations:** 1 Department of Sport Science, Faculty of Social Sciences, Linnaeus University, SE-391 82 Kalmar, Sweden; 2 Department of Health Sciences, Lund University, Lund, Sweden; 3 Clinical and Molecular Osteoporosis Research Unit, Department of Orthopedics and Clinical Sciences, Skåne University Hospital, Lund University, Malmö, Sweden; 4 Faculty of Education and Society, Department of Sport Sciences, Malmö University, Malmö, Sweden; 5 Department of Translational Medicine, Lund University, Malmö, Sweden; 6 Department of Medical Imaging and Physiology, Skåne University Hospital, Malmö, Sweden

**Keywords:** Postural orientation errors, Functional performance, Sex difference, Lean body mass

## Abstract

**Background:**

Studies investigating postural orientation in uninjured youth athletes are scarce. Understanding how postural orientation during functional performance tests change with age in uninjured athletes has the potential to enhance awareness of changes in performance after injury and to set realistic goals for injured athletes. Thus, the aim of this study was to explore postural orientation during functional tasks at early adolescence, and changes in postural orientation from early to middle adolescence and relate this to sex, type of sport and right leg lean body mass (RLLBM).

**Methods:**

In this cohort study 144 (38% female) youth athletes (mean age 13.5 years, SD 0.3) were included at baseline and 86 of these at follow up 2 years later. Four functional performance tests were visually evaluated for Postural Orientation Errors (POEs) with an ordinal scale, ranging from 0 (good) to 2 (poor), yielding a maximum total POE score of 51, and RLLBM by dual energy X-ray absorptiometry.

**Results:**

Improvements were observed in the total POE score from baseline to follow-up, median difference − 10 and − 7 (*p* < 0.001) for female and male athletes, respectively. At follow-up, female athletes had lower total POE score (median 18) than males (median 24) (*p* = 0.01). There were no differences in POE scores between sports type (team, individual, aesthetic) (*p* = 0.20–0.98) and no relationship between total POE score and RLLBM (r_s_ = 0.09, *p* = 0.42).

**Conclusions:**

POEs appear to be quite common in young athletic population, but improvements are achieved over time. At mid-adolescence, female athletes seem to have less POEs than males. Neither sport type nor RLLBM seem to influence postural orientation.

## Background

Functional performance tests for assessment of sport performance often include tests of muscle strength, endurance and/or power [1–3] and in some cases movement quality [4, 5]. However, most of the studies [4, 5] on movement quality are performed on young adults and studies on movement quality in youth athletes are scarce [6]. Functional and sport performance gradually improve with maturation [7] and full development of a specific skill depends on full maturation of the nervous system [8]. During maturation there are progressive changes in muscle mass, power and strength, which are of importance for the improvement of physical performance [9]. However, these developments vary between sexes with general greater improvements for boys [9–11] that continues to advance during adolescence compared to girls who reach an earlier plateau [9]. Hence, an important factor to consider when examining youth athletes is their sex and age because this may influence their functional performance, including quality of movement.

Postural orientation is one aspect of movement quality, which is defined as the ability to maintain an appropriate relationship between the body segments and between the body and the environment when performing a task [12]. Postural orientation is one component that, together with postural stability, constitutes postural control [13]. Postural control has been noted to be important for performance in several sports [14–16]. For example, soccer players have been found to have better postural control, in terms of less postural sway, compared to participants involved in limited contact sport or no sport at all [14]. However, to our knowledge, research on the relationship between postural orientation and sport performance seem to be lacking.

For postural orientation of the lower extremity, the knee joint is often described as normal (aligned), varus, or valgus [17] where valgus has been highlighted as a factor associated with knee injury [18–20]. For example, some anterior cruciate ligament (ACL) tears in sports involve a noncontact mechanism, with the lower extremity displaying a dynamic knee valgus moment at the impact of injury [18]. Furthermore, patients with ACL injury seem to have different lower limb biomechanics [21], in addition to poorer trunk control [22] and poorer postural stability [13], compared with healthy controls. However, the interpretation of data from these studies in a clinical setting without any reference values from an uninjured population is challenging.

From a sport medical perspective, reference values from age-matched athletic controls without injury are important to identify any abnormal and/or impaired values when testing groups of patients. To our knowledge, there is only one study [23] investigating postural orientation during a functional performance task in healthy children and adolescence (from 9 to 16 years of age). In that study, no differences between sexes in absolute values were noted but a different effect of age for boys and girls [23]. However, only one dynamic test, the single-limb mini squat, was performed to assess postural orientation and only the medio-lateral knee position was analyzed [23]. In addition, no data on sport participation were specified for the participants in the study, thus, it was unclear whether they were athletes. Thus, studies investigating postural orientation in youth athletes without injury are warranted.

Understanding how postural orientation during functional performance tests change with age in uninjured athletes has the potential to enhance awareness of changes in performance after injury and to set realistic goals for injured athletes. The knowledge from the present study could help sport physical therapists, coaches and/or athletic trainers when assessing functional performance in young athletes.

The aims of this study were to explore: 1) postural orientation during functional tasks at early adolescence (baseline assessment); 2) any changes in postural orientation from early to middle adolescence (baseline to follow-up 2 years later); 3) any sex differences in postural orientation; 4) differences in postural orientation between different sports; and 5) the relation between postural orientation and lean body mass, in youth athletes.

## Methods

### Study design

Data for this cohort study, following to the STROBE statement [24], were collected during 2013–2017 as part of the Malmö Youth Sport Study (MYSS), in detail described in previous publications [25, 26]. In summary, MYSS is an ongoing longitudinal cohort study, including boys and girls (later young men and women), investigating physiological, psychological and social factors associated with sports performance, academic success and long-term physical activity [25]. The participants in the MYSS project are young athletes attending a sport school in the southern part of Sweden, a part of the Swedish National Sport Education program, aiming for an elite career. The selection criteria for acceptance to the school are sport merits and one of the aims of the education is sport talent development. The school provides organized sport specific training during school hours allowing students to combine educational work with sports. The students practice their sport during school hours (≥450 min/week) outside regular training and competitions after school.

### Participants and procedure

**Fig. 1:**
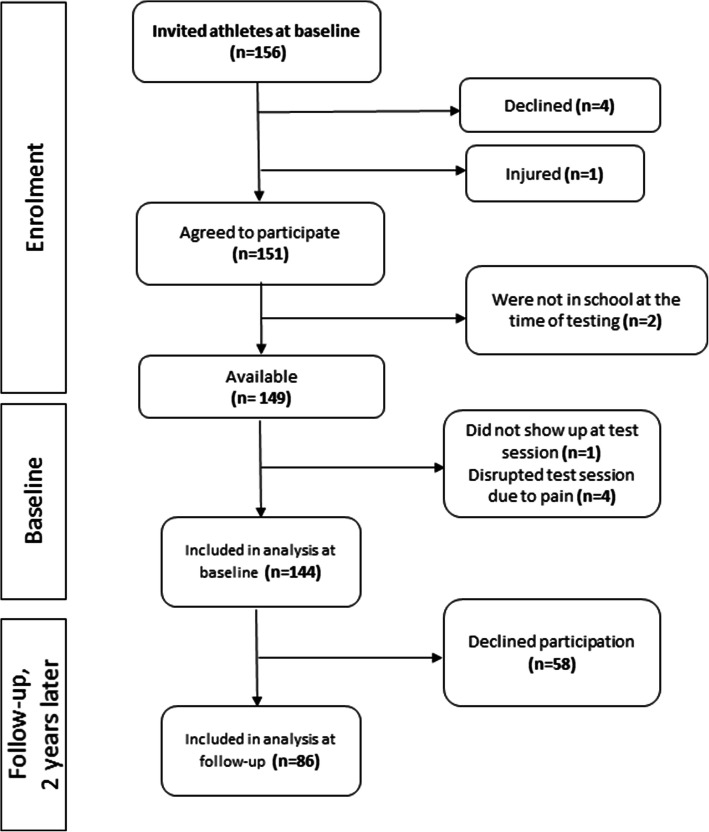
Flow chart of inclusion process of athletes for the evaluation of postural orientation errors (POEs)

In this report, we included athletes who were involved in team sports (soccer, ice hockey, floorball and basketball), individual sports (swimming, athletics, tennis, badminton and squash), or aesthetic sports (diving, figure skating and artistic gymnastics). From the total cohort of the MYSS project (*n* = 156), 144 (38% girls) healthy adolescent athletes were included. Thirteen-year old athletes were assessed at baseline, during the winter months (2013–2014 and 2014–2015), (Fig. 1, Table 1) and, of these, 86 were assessed approximately 2 years later at the same season and with the same test battery. Their mean (SD) age was 13.5 (0.3) years at baseline and 15.6 (0.3) years at follow up.

**Table 1:** Athletes’ characteristics for female and male athletes at baseline (*n* = 144) and at follow-up (*n* = 86)

Anthropometrics	Male athletes at baseline (***n*** = 90)	Male athletes at follow-up (***n*** = 52)	Female athletes at baseline (***n*** = 54)	Female athletes at follow-up (***n*** = 34)
	Mean (SD)	Mean (SD)	Mean (SD)	Mean (SD)
Height (cm)	163 (10)	176 (8)	161 (6)	166 (6)
Weight (kg)	51 (10)	65 (10)	52 (6)	60 (6)
TBLBM (% of bodyweight)	78 (0.6)	80 (0.4)	71 (0.4)	68 (0.4)
**Main Sport**	**n**	**n**	**n**	**n**
Football ^a^	41	26	17	9
Ice hockey ^a^	11	8	5	4
Basketball ^a^	9	2	6	4
Floorball ^a^	5	3	6	4
Swimming ^b^	7	3	3	2
Athletics ^b^	0	0	7	4
Tennis ^b^	7	5	2	1
Squash ^b^	1	1	0	0
Badminton ^b^	4	1	0	0
Diving ^c^	1	0	4	3
Figure skating ^c^	1	1	4	3
Artistic gymnastics ^c^	3	2	0	0
**Type of sports**	**n**	**n**	**n**	**n**
Team ^a^	66	39	34	21
Individual ^b^	20	10	16	10
Aesthetic^c^	4	3	4	3

*TBLBM* Total body lean body mass

^a^Team sports

^b^Individual sports

^c^Aesthetic sports

Trained physiotherapists collected data, with video-recording, for functional performance tests at the athletes’ school. Anthropometrics (height, weight and total body lean body mass (TBLBM) were measured in a laboratory setting at another occasion within 1–3 months from the functional performance test session. Athletes with any difficulty moving around on the day of testing, or reports of lower extremity injury, limiting the completion of the tasks, were excluded (*n* = 1).

### Measurements of postural orientation errors

The athlete’s performance during 4 functional performance tests, previously described by Nae et al. [27, 28], was videotaped, using a digital camcorder (1920 × 1080 pixels; 30 Hz; Everio GZ-HM650BE; JVC, Yokohama, Japan) placed on a tripod in front of the athlete, perpendicular to the frontal plane, for later assessment of Postural Orientation Errors (POEs). To ensure that the whole movement was captured during testing, the camera was positioned 2–4 m (m) in front of the athlete in line with his or her waistline (approx. 1 m off the floor).

Athletes were instructed to wear shorts and a tight top. The first test (single-leg mini squat) was performed barefoot, whereas in the remaining tests athletic shoes were worn. All athletes performed the tests in the order that they are described below, starting on the right leg. Prior to each test, the test leader gave standardized instructions along with a visual demonstration of the test. The athlete was allowed 2–3 practice trials per test, or per side for the one-legged test, before initiating the testing. For the Drop-jump, practice trials were given until the athlete was familiarized with the procedure.

#### Single-leg mini squat

For the Single-leg mini squat (SLS), the athlete was standing with the arms alongside the body on one leg and with the second toe placed on a longitudinal line. The athlete was asked to bend his/her knee, without bending forward from the hip, until he/she no longer could see the line along the toes (corresponding to about 50 degrees of knee flexion), and then return to extension. The test was repeated 5 times on each leg and POEs were assessed during the entire movement, from starting position through return to this same position.

#### Forward lunge

For the Forward Lunge (FL), the athlete was standing with the arms alongside the body and with feet hip-width apart on the floor. The athlete took a long stride forward, about 1 m, flexed the knee to approximately 90°, and pushed back to starting position by extending the front leg. The test was repeated 5 times on each leg and the front leg was assessed in the landing phase from initial contact until maximum flexion of the knee.

#### Drop-jump

The Drop-jump (DJ) test was performed with the athlete standing on a step board, approximately 30 cm high, with feet hip-width apart. The athlete dropped from the step-board with both feet leaving the box simultaneously, then performed a maximal vertical jump upon landing. Arm swing was allowed during the jump and the jump was repeated 3 times. The POEs were assessed during the first landing, from first contact with the floor to extended knees.

#### Single-leg hop for distance

For the Single-leg hop for distance (SLHD), the athlete was standing on one leg, with the other leg lifted from the floor by flexing the knee. The athlete jumped forward as far as possible, taking off and landing on same foot with a safe and controlled landing maintaining balance on landing for 2 to 3 s. Arm swing was allowed during the jump. The test was repeated 3 times and POEs were assessed during landing, from first contact with the floor to extended knee.

### Scoring of postural orientation errors

A trained physical therapist (SRA) observed and rated POEs from the video recordings according to a previously evaluated protocol [28]. POEs were assessed by evaluating 1) pronation of the foot (SLS only), 2) knee medial-to-foot position (KMFP), 3) femoral valgus, 4) deviation of pelvis in any plane (lateral deviation, tilt and/or rotation of pelvis) and 5) deviation of trunk in any plane (forward, lateral and/or rotation) as described [28].

A 3-point ordinal scale, ranging from 0 to 2, was used for the evaluations, with 0 indicating good postural orientation (no signs of POEs), 1 fair (minimal signs of POEs), and 2 poor (clear signs of POEs). When the execution of the test did not have any similarities to the test a score of 3 was given, representing very poor postural orientation, thus the maximum within-task POE score was given [28]. A POE was scored as fair or poor when it occurred at least 3 out of 5 times in the tasks performed with 5 repetitions and at least 2 out of 3 times in the tasks performed with 3 repetitions (Table 2). Both the within-tasks POE score (the sum of all POEs within a task), and the total POE score were calculated to a percentage scale (0–100) and used in the analyses (Table 2).

**Table 2:** Tasks, POEs within each task, and calculations for the percentage scale (within-task and total POE scores)

Functional Task	Foot Pronation	KMFP	Femoral valgus	Pelvis segment	Trunk Segment	Within-Task POE Score
Single-leg mini-squat	X	X	X	X	X	$\frac{\mathrm{sum}\ \mathrm{score}}{15}x\ 100$
Forward lunge		X	X	X	X	$\frac{\mathrm{sum}\ \mathrm{score}}{12}x\ 100$
Drop Jump		X	X	X	X	$\frac{\mathrm{sum}\ \mathrm{score}}{12}x\ 100$
Single-leg hop for distance		X	X	X	X	$\frac{\mathrm{sum}\ \mathrm{score}}{12}x\ 100$
Total POE score						$\frac{\mathrm{sum}\ \mathrm{score}}{51}x\ 100$

*KMFP* knee medial to the foot position

### Reliability analysis

Intra-rater and inter-rater reliability were evaluated from 10 athletes’ video recordings for within-task POE score and total POE score. Inter-rater reliability was assessed by two authors (SRA and JN) for each within-task POE score with Cohen’s kappa [29, 30] and showed moderate to almost perfect agreement (kappa values from 0.74 to 0.88, *p* ≤ 0.0001), according to Landis and Koch [31]. Intra-rater reliability, analyzed on two separate occasions within 2 weeks, for each within-task POE (assessed by the author SRA) was calculated with intra-class correlation coefficient (ICC_2,1_), with the two-way random effect model (absolute agreement definition, 95% confidence intervals (CI)) and indicated excellent agreement (ICC_2,1_ value from 0.824 to 0.98, *p* ≤ 0.002). Inter-rater reliability for total POE score showed excellent agreement (Cohen’s kappa value 0.875, *p* < 0.001). A Wilcoxon’s rank test was also calculated, revealing no systematic difference between raters for the total POE score (*p* = 0.32). Total POE score for intra-rater reliability, assessed with ICC_2,1_, was 0.95 (CI: 0.82–0.99, *p <* 0.001).

### Anthropometry

Body height (cm) was measured, with a Holtain Stadiometer (Holtain LTD, Pembrokeshire, UK) and body mass (kg) with an electric scale (Avery Berkel HL 120 Electric Scale, Avery Berkel, West Midlands, UK). Total body lean body mass (TBLBM) and right leg lean body mass (RLLBM) was measured by dual energy X-ray absorptiometry (DXA) (iDXA® version enCore 13.60, Lunar Corporation, Madison, WI, USA). When estimating TBLBM and RLLBM we used a total body scan and standard adult software. The measurements were done with the participants non fasting, dressed in light clothes, with no shoes, and with the athletes in a supine position according to standard procedure recommended by the manufacturer. Two trained research technicians performed all measurements and software analyses. All measurements were done within 1–3 months from the functional performance test session. The DXA apparatus was calibrated daily, by use of a phantom. Coefficient of variation (CV%) was for TBLBM 0.6% .

### Statistical methods

Statistics were calculated using IBM SPSS (IBM SPSS Statistics for Windows, Version 23.0. IBM, Armonk, NY). Descriptive data are presented with median and quartiles for categorical data, while means and standard deviation (SD) were used to describe continuous data. A small, likely non-clinically relevant, difference was found between the right (median 3, quartiles 1–4) and left legs (median 2, quartiles 2–3) in the DJ test (*p* = 0.002). No other differences were observed between the right and left legs; therefore, data were analyzed for the right leg only. For drop-out analysis, demographic data (height and weight) and baseline screening results are presented for the drop-outs (those who did not attend at follow-up, *n* = 58) and the participants (included in the follow-up analysis, *n* = 86). Males and females were analyzed separately except for the comparison between sports type (team, individual, aesthetic sports). For comparison baseline vs follows up, data were analyzed with the Wilcoxon’s rank test for POEs and with the Paired-sample t-test, with 95% CI, for RLLBM. TBLBM value is expressed in kg and percentage of body weight together with relative to bodyweight (rTBLBM). The Mann-Whitney U test was used for comparison between sexes, and the Kruskal-Wallis H for comparison between sports type. Differences between POE score for different tests were analyzed with Friedman Test and Wilcoxon’s rank test. Fisher’s exact test was used to assess any differences in the distribution of males and females between the groups of different sports type. The Spearman’s correlation coefficient was used to analyze the association between changes in POEs (median difference baseline vs follow-up) and changes in RLLBM (mean difference baseline vs follow-up). The level of significance was set at *p* ≤ 0.05.

## Results

### Participants – drop-out analysis

A total of 144 athletes were screened at baseline and, of these, 86 athletes were included in the follow-up analysis. At baseline, the total POE score was 29 (q1–3 = 12) for the drop-outs (*n* = 58) and 31 (q1–3 = 13.5) for the follow-up participants (*n* = 86). Table 3 gives the baseline values for total POE score, weight, and height at baseline for female drop-outs (*n* = 20) and follow-up participants (*n* = 34) and male drop-outs (*n* = 38) and follow-up participants (*n* = 52).

**Table 3:** Total POE score, presented with median together with q1–3, height (cm) and weight (kg) presented with mean (SD) for drop-outs and follow-up participants (FUP)

	Female drop-outs (***n*** = 20)	Female FUP (***n*** = 34)	Male drop-outs (***n*** = 38)	Male FUP (***n*** = 52)
**Total POE score**	26 (8.5)	31 (15.5)	31 (12)	31 (12)
**Height (cm)**	162 (7)	162 (6)	164 (10)	164 (9)
**Weight (kg)**	52 (6)	52 (6)	52 (11)	50 (9)

### Baseline assessments of postural orientation errors

Within-task POE scores for each task and total POE score, at baseline, are given in Table 4 for female and male athletes as well as POE scores according to sports type. Median POE score in SLS and SLHD were significantly higher compared to FL and DJ for both females and males (*p* < 0.001).

**Table 4:** Within-task POE score for the single-leg mini squat (SLS), forward lunge (FL), drop jump (DJ) and single leg hop for distance (SLHD), and total POE score, are presented for right leg, female and male athletes and for different sports type (team, individual, aesthetic) at baseline (*n* = 144). Values are median (quartiles, minimum-maximum)

Task/POE	Females (***n*** = 54)	Males (***n*** = 90)	Team (***n*** = 100; female ***n*** = 34)	Individual (***n*** = 36; female ***n*** = 16)	Aesthetic (***n*** = 8; female ***n*** = 4)
**SLS**	33 (20;40, 13;60)	33 (20;47, 13;73)	33 (20;47, 13;73)	33 (20;45.25, 13;60)	33 (21.75;42.25, 13;53)
**FL**	25 (17;42, 0;58)	25 (17;33, 0;58)	25 (17;39.75, 0;58)	25 (17;33, 0;58)	12,5 (8;39.75, 0;50)
**DJ**	25 (17;22, 0;58)	25 (8;33, 0;67)	25 (10.25;33, 0;67)	17 (8;31, 0;42)	25 (2;31, 0;50)
**SLHD**	33 (25;44, 8;75)	33 (25;58, 17;83)	33 (25;50, 17;83)	33 (25;56, 17;75)	29 (21;51.75, 8;58)
**Total POE score**	28 (24;35.5, 10;53)	31 (25;37, 12;55)	31 (25;37, 14;55)	26 (24;32.5, 10;53)	27 (21.25;33.5, 12;53)

### Changes in postural orientation errors over time

There were significant improvements in the total POE score between baseline and follow-up for both female (*p <* 0.0001) and male athletes (*p <* 0.0001) (Table 5). There were also improvements in all tests (SLS, *p* = 0.001; FL, *p* < 0.001; DJ, *p <* 0.001; SLHD, *p* = 0.024) for females and in FL (*p <* 0.001), DJ (*p <* 0.001) and SLHD (*p =* 0.001) for males.

**Table 5:** Within-task POE score for the single-leg mini squat (SLS), forward lunge (FL), drop jump (DJ) and single leg hop for distance (SLHD) and total POE score, and differences between baseline and follow-up for female (*n* = 34) and male (*n* = 52) athletes. Values are median and quartiles

Task/POE	Baseline		Follow-up		Baseline vs follow-up	
	Females (***n =*** 34)	Males (***n =*** 52)	Females (***n =*** 34)	Males (***n =*** 52)	Females (***n =*** 34)	Males (***n =*** 52)
**SLS**	33 (20;40)	33 (20;47)	20 (13;28.5)	33 (20;40)	−7 (−0;6.25)^a^,^b^	0 (−14;7)^b^
**FL**	25 (17;42)	25 (17;33)	8 (7;17)	17 (8;17)	−17 (−33;9)^a^	− 16 (−25;0)^a^
**DJ**	25 (17;425)	17 (8;33)	8 (0;25)	8 (0;23)	−16.5 (−25;0)^a^	−8 (− 16;0)^a^
**SLHD**	33 (25;50)	42 (25–58)	29 (17;42)	33 (25;42)	−8 (−19;8)^a^	− 8 (− 17;0)^a^
**Total POE score**	31 (24;39.5)	31 (25;37)	18 (12;25.5)	24 (18.5;31)	−10 (−18;-4)^a,b^	−7 (−1.5;-2)^a,b^

^a^significant difference between baseline and follow-up (*p* ≤ 0.007)

^b^significant difference between sexes (*p* ≤ 0.01).

### Sex differences

At baseline, no differences were found between males and females for any POE scores (*p* = 0.06–0.42). At follow-up, female athletes scored better in the SLS test (*p* = 0.004) and had lower total POE score than males (*p* = 0.01) (Table 5).

### Postural orientation errors in different sports type

POE scores according to sports type are presented in Table 6. There were no differences in sex distribution between the groups of different sports type (*p* = 0.40). No differences in POE scores were found between sports type (team, individual, aesthetic) at baseline (*p* = 0.20–0.98) whereas aesthetic athletes performed significantly better in SLS at follow-up compared to team athletes (*p* = 0.02). All groups significantly improved Total POE score (*p* = 0.0001–0.04).

**Table 6:** Within-task POE score for the SLS, Lunges, DJ and SLHD, and total POE score, given for the different sports type at baseline and follow-up (*n* = 86). Values are median and quartiles

Task/POE	Baseline			Follow-up			Baseline vs Follow-up		
	Team (***n =*** 60; female ***n*** = 21)	Individual (***n =*** 20; female ***n =*** 10)	Aesthetic (***n =*** 6; female ***n =*** 3)	Team (***n =*** 60)	Individual (***n =*** 20)	Aesthetic (***n =*** 6)	Team (***n =*** 60)	Individual (***n =*** 20)	Aesthetic (***n =*** 6)
**SLS**	33 (20;70)	33 (20;42.25)	33 (23.5;41.75)	33 (20;40)	23.5 (13;38.25)	16.5 (11.5;21.75) ^b^	0 (−14;7)	−7 (−20;5.25)^b^	−20 (−21.75;4.5)^a,b^
**Lunges**	25 (17;42)	25 (10.25;33)	12.5 (6;44)	12.5 (8;17)	8 (0;17)	12.5 (0;37.5)	−17 (− 25;8)^b^	−16.5 (−31;0)^b^	8,5 (−23.25;19)
**DJ**	25 (10.25;33)	17 (8;33)	25 (18.75;37.25)	8 (0;25)	17 (2;31)	8 (6;10.25)	−8 (− 25;0)^b^	0 (−14.25;6)	−17 (−27;12.75)^b^
**SLHD**	42 (27;58)	33 (19;56)	25 (20.75;39.25)	33 (25;42)	25 (17;33)	17 (15.75;44)	−8 (−17;0)^b^	0 (− 25;0)^b^	− 8 (−12.25;2.5)
**Total POE score**	31 (25;39)	25 (24;31)	27 (18;37)	24 (16;29)	19 (14;29.5)	15 (9.5;28)	−8 (−15.5;-4)^b^	−6 (−16;-0.5)^b^	−7 (− 18.75;-1.50)^b^

*SLS* single-leg mini squat, *FL* forward lunge, *DJ* drop jump, *SLHD* single leg hop for distance

^a^significant difference between groups (*p* = 0.032)

^b^significant difference between baseline and follow-up (*p* ≤ 0.042)

### Lean body mass and the association between POEs and RLLBM

**Fig. 2:**
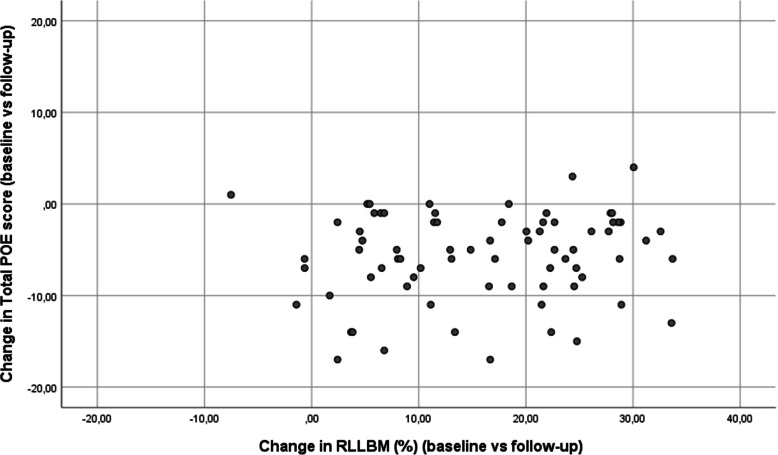
Scatter-plot of change in total POE score and RLLBM (%) in youth athletes (*n* = 83) (r_s_ = 0.15, *p* = 0.18)

There was a significant increase of 12 kg (95% CI 11 to 13, *p* < 0.001) in TBLBM for the male athletes (*n* = 52), from baseline to follow-up, corresponding to an rTBLBM of 2% (95% CI 1 to 3) increase. RLLBM increased with 2 kg, from 6.8 to 8.9 kg, (95% CI 1.8 to 2.3, *p* < 0.001). For the female athletes (*n* = 34), the TBLBM had increased by 4 kg (95% CI 3 to 5, *p <* 0.001) but decreased in rTBLBM by 3% (95% CI =2 to 4, *p <* 0.001) from baseline to follow-up. RLLBM increased with 0.8 kg, from 6.3 to 7.1 kg, (95% CI 0.5 to 0.9, *p <* 0.001). No relationship was found between the total POE score difference (baseline vs follow-up) and RLLBM difference (baseline vs follow-up) for male (r_s_ = 0.15, *p* = 0.31) or female athletes (r_s_ = 0.436, *p* = 0.482), or for the total cohort (r_s_ = 0.09, *p* = 0.42) (Fig. 2).

## Discussion

The main observation in this study was that POEs seem to be quite common in early adolescent athletes. Both female and male athletes demonstrated rather high POE scores, indicating poor postural orientation, at age 13 with no differences between females and males (*p* ≥ 0.06). Thus, appropriate postural orientation may not be expected in this young population. When examined 2 years later, both female and male athletes improved their total POE score between baseline and follow-up but female athletes scored significantly better in the total POE score (*p* = 0.012). Neither sport type nor LBM was associated with POE scores.

The total POE score (28 and 31 for females and males, respectively) at the age of 13, noted in the present study, are higher than POE scores noted in women (26) and men (20.5) with ACL injury (mean (SD) age 26.7 (6.5) [32]. However, in the study on ACL injured participants, the test battery consisted of five test of functional task and six segment-specific POEs yielding a higher maximum total POE score of 73. The median POE score for *FL* and *DJ* test was 25 respectively and 33 for both the *SLS* and *SLHD* test. This indicates that, at early adolescence, we can expect POEs to a fairly large extent in functional tasks, which is important knowledge for professions that examine athletes in different aspects of neuromuscular control. The POE scores for the *SLS* and the *SLHD* was higher than for the *FL* and *DJ*, indicating that the *SLS* and *SLHD* may better detect POEs in this young athletic population compared to *FL* and *DJ*. Further, the higher scores for the *SLS* and the *SLHD* suggests that unilateral tests are, not surprisingly, more demanding for maintaining appropriate postural orientation than two-legged tests. One advantage of single-leg tests is their ability to detect between-limb imbalances [33] and thus, might be more useful when aiming to detect postural orientation errors between injured and non-injured limb.

The significant improvements in the total POE score between baseline and follow-up could be related to natural neuromuscular improvements from early to mid-adolescence. In a previous study on youth tennis players, the authors found large age effects on neuromuscular lower-limb asymmetries (between-limb differences) [33]. However, contrary results have been found in other investigations [10, 34]. In a study on elite male youth soccer players, the stage of maturation did not show any effect on the level of asymmetry, in functional performance tasks, in terms of landing force and between-limb difference [34]. In another study [10], investigating neuromuscular control on 1140 youth athletes, no age effects, from 9 to 17 years, were noted in limb alignment measured as medial knee displacement during a drop-jump. Although there was an overall performance enhancement in the current study, the median POE scores for the SLS (20 for females, 33 for males) and SLHD (29 for females, 33 for males) were still rather high at follow-up, suggesting that POEs are still present at mid-adolescence. Yet, there were relatively large improvements for FL and DJ for females (− 17 and − 16.5, respectively) and in FL for males (− 17) compared to the improvements noted in the SLS and SLHD tests (0 to − 8). Hence, at mid-adolescence, the use of single-leg tests might be more suitable to detect postural orientation errors. Taken together, improvements in the total POE score between baseline and follow-up were evident for both female and male athletes suggesting some kind of maturity effect.

In the present study, we did not find any sex differences in POEs at the age of 13. Previous have reported that young girls seem to have better postural stability [35] and less body sway than boys [36]. However, these studies included younger non-athlete children, 8–12 and 3–6 years of age, respectively, and measured postural stability (measured as motion of the center of pressure) and not postural orientation. In the present study, female athletes scored significantly better in total POE score than males at mid-adolescence. One possible explanation for the sex differences at mid-adolescence could be that females mature earlier than males [37] and thus have reached a more developed motor control system. However, we can only speculate regarding the impact of maturity level as no maturity data were collected.

No differences in POE scores were found between sports type (team, individual, aesthetic) at baseline whereas aesthetic athletes performed significantly better in SLS at follow-up compared to team athletes. In addition, there were no differences in sex distribution between groups and all sport groups significantly improved in the total POE score. The aesthetic group of athletes included diving, artistic gymnastic and figure skating. It might be that these athletes improve functional performance, including awareness of body alignment, within their sports as it has been demonstrated that sport skill has an impact on balance ability [38, 39]. Nevertheless, due to the small sample size of aesthetic athletes (*n* = 6) in the present study, further studies are needed on the possible association between sport type and postural orientation.

There was a significant increase of 12 kg in TBLBM for the male athletes, from baseline to follow-up, corresponding to an increase in rTBLBM of 2%. Although female athletes’ TBLBM also increased (4 kg), the rTBLBM had decreased by 3% from baseline. The fact that males obtain greater amounts of muscle mass, whereas females gain significantly more fat mass, has previously been reported [40]. In addition, whereas no sex differences in muscle strength seem to exist before the age of 14, male athletes have a much greater muscle strength development from age 14 to 17 compared to female athletes [10]. However, in our study, no strength data are available, and the development of muscle mass did not seem to influence postural orientation as no relationship was found between the total POE score and RLLBM. Thus, the improvement in POE scores, baseline to follow-up, may be affected by other factors than development of muscle mass, such as neural adaptations to training and natural maturity of the nervous system. Further studies may investigate if muscle strength and state of maturity influence postural orientation.

### Strengths and limitations

This study is the first to provide values of postural orientation and changes over time in youth athletes, related to age, sex and sport type. Another strength is the use of a reliable and valid clinically applicable scoring protocol for assessing POEs. Yet, some limitations in our study need to be recognized. First, the values presented in the present study can only be applied to young athletes aged 13–16 years and cannot be generalized to the general population of the same age. In addition, only 86 athletes, of the 144 at baseline, were evaluated at the 2-year follow-up. We cannot exclude that the drop-outs could have affected the result, although we observed no clinically relevant differences between follow-up participants and drop-outs in baseline total POE score, or characteristics (weight, height). Another limitation is that no definite maturation evaluation, except from age and TBLBM, or muscle strength assessment was performed. In addition, although injured athletes, at the time of testing, were excluded from participating, we had no data as to whether the included athletes had sustained any previous injuries. Although declared healthy, previous injury might effect physical performance long after onset [41]. Lastly, as there were few aesthetic athletes (*n* = 6) in the present study, a larger sample size is desirable in future investigations to explore any differences in POEs between type of sports.

## Conclusion

Postural orientation errors appear to be quite common in a young athletic population, although improvements were noted from early to mid-adolescence, particularly among females. At early adolescence, there seems to be no sex differences in postural orientation, whereas female athletes may perform better in some functional tests at mid-adolescence. Further, differences between types of sports could not be demonstrated in the present study and the lack of relation between postural orientation and lean body mass indicates that the amount of muscle mass does not seem to influence postural orientation.

## Data Availability

The data collected and analyzed in the current study are not publicly available due to ethical restrictions, but are available from the corresponding author upon reasonable request.

## Abbreviations

POEs: Postural Orientation Errors
SLS: Single-leg mini squat
FL: Forward lunge
DJ: Drop jump
SLHD: Single leg hop for distance
FUP: Follow-up
TBLBM: Total body lean body mass
rTBLBM: Relative Total body lean body mass
RLLBM: Right leg lean body mass
CI: Confidence intervals
SD: Standard deviations
